# Transitions in adolescent boys and young Men’s high-risk sexual behaviour in India

**DOI:** 10.1186/s12889-020-09191-6

**Published:** 2020-07-11

**Authors:** Santosh Kumar Sharma, Deepanjali Vishwakarma

**Affiliations:** 1 International Institute for Population Sciences, Deonar, Govandi Station Road, Mumbai, Maharashtra 400088 India; 2 Deepanjali Vishwakarma, Doctoral Fellow, International Institute for Population Sciences, Deonar, Mumbai, India

**Keywords:** Adolescents, High-risk sexual behaviour, Multiple sexual partner, Relationship, Condom use, Alcohol

## Abstract

**Background:**

The sexual behaviour of adolescents is of importance due to the engagement in risky sexual activity at a too early age, which may be associated with the adverse outcomes. The study aims to understand the transitions in adolescent boys and young men’s high-risk sexual behaviour in India using two rounds of Indian demographic health survey, NFHS-3 (2005–06) and NFHS-4 (2015–16).

**Methods:**

A total of 25,538 in NFHS-3 (2005–06) and 35,112 in NFHS-4 (2015–16) men were considered for the analysis. Men have been divided into two age groups as 15–19 years (adolescent) and 20–24 (young men) for comparison purposes. Descriptive and multivariate statistics have been used.

**Results:**

Overall, high-risk sexual behaviour has increased among adolescent boys (64 to 70%) and young men (18 to 27%) from 2005–06 to 2015–16. The trend of live-in relationship has increased among adolescent boys of rural areas (0.6 to 6.0%) as well as in urban areas (3.1 to 10.9%) over the last 10 years. Adolescent boys having 10th and above years of schooling (AOR = 1.98; *p* < 0.01), residing in urban areas (AOR = 2.23; *p* < 0.01), and belonging to the affluent class of households (AOR = 1.41; *p* < 0.05) were more likely to engage in high-risk sexual activity than the young men in India. The odds of high-risk sexual behaviour was higher among alcohol-using adolescent boys (AOR = 1.82; *p* < 0.01) and young men (AOR = 2.38; *p* < 0.01) in 2015–16.

**Conclusions:**

The study concludes that early sexual debut, lower prevalence of condom use at first sexual experience, tendency of live-in-relationship, and alcohol consumption indicate the hazardous interconnection between such behaviours among adolescent boys over the last decade which placed them at higher-risky sexual behaviour as compared to young men. Adolescent’ sexual behaviours have both short-term and long-term consequences, and interventions that focus on multiple domains of risk may be the most effective in helping to promote broad reproductive health among young adults.

## Background

Adolescent in India comprises almost 22% of India’s population, and their number is only going to increase in the future [1, 2]. They are growing up in an environment that is very different from their parents in which they grew up. The sexual conduct of adolescents is of significance because of the expanding number of sexually active adolescents comprehensively [3, 4]. While the commencement of sexual action is a piece of typical conduct and advancement, it might likewise be related with antagonistic results, particularly when sexual conduct includes commitment in sexual activity at a too early age, or regardless of the risk involved [5]. Adolescence is a period of transition when an individual’s personality develops which includes his/her masculinity/femininity social, cultural, economic and biological events take place, which set the stage for adulthood [2].

The traditional Indian society has been allied with illustration of sexuality and sexual liberalism in the form of art. The first text to contemplate sexual expression as a science was Kamasutra, which originated before the sixth century [6]. Regardless of even the old content of Indian religious philosophy perceiving the principal idea of puberty and pushing unambiguous implicit rules for the stage, the conception of adolescence is moderately new in current India, and the adolescent has open space in strategy plans or policy formulations.

In modern India, age limits of adolescents varies from program to program or policy to achieve their goals. According to the National Youth Policy of India, adolescents is defined as the age group 13–19 years; while, the Reproductive and Child Health program makes reference to adolescents as being between 10 and 19 years old. The Juvenile Justice (Care and Protection) Act (JJ Act) 2000 and now the JJ Act 2015, and the Protection of Children from Sexual Offenses Act (POCSO Act), 2012 characterize all people up to the age of 18 years as adolescents [7].

There are many factors which encompasses by adolescent’s sexuality such as developing intimate partner relationships, gender identity, sexual orientation, religion and culture [8]. Sexual behaviour perhaps influenced by many factors like physiological cultural and social pressures, which vary from generation to generation [3]. Sexual exposure during adolescence is a matter of grave concern due to the risk of transmission of sexually transmitted infections, including HIV infection/AIDS, teenage pregnancy, and adolescent fatherhood [9, 10]. Early sexual introduction prompting HIV infection involves extraordinary concern in many developing, as well as underdeveloped countries [11]. Joshi and Chauhan [12] stated that there is a high level of premarital and unsafe sexual behaviour among young individuals in India. A study conducted by Sharma [13] on adolescents and youth in low income slums of Mumbai articulated that the phase of adolescence is also marked by the experimentation and influence of friends and peer groups. During this adolescent’s age, they begin investing more time exterior the home, getting away the guardian ship of the adult members of their families. Adolescents and young individuals often get fascinated by mass media, friends, and peer pressure without sufficient knowledge of prevention, which inspires them to indulge in hazardous ways of life such as smoking, alcohol or drug use, and sexual activity. As a consequence, the hazard of sexually transmitted diseases (STDs) and HIV/AIDS may be noteworthy among adolescents and young individuals [14]. Numerous studies from other countries evident that there are number of factors such as multiple partner, relationships, and family factors, associated with condom use among young individuals [15–17]. At the individual level, condom use among youth is positively correlated with several factors such as schooling, self-worth, awareness about the advantageous of condom use, anticipated infection risk, and socio-economic status of households, whereas there is negative association between sexual debut at early age and substance use [17–19].

In view of the above, the study was undertaken to understand the transitions in adolescent boys and young men’s high-risk sexual behaviour in India using the preceding two rounds of Indian demographic health survey, NFHS-3 (2005–06) and NFHS-4 (2015–16). The specific objectives of the study are to understand the changes in risky sexual behaviour and safe sexual practices at first and last sexual experience among adolescent boys and young men in India over the last decade, and to determine the factors associated with risky sexual behaviour among adolescent boys (15–19) and young men (20–24) in India.

## Methods

The analysis of this study is based on two rounds of National Family Health Survey (NFHS) survey, an Indian variant of Demographic and Health Surveys (DHS), conducted during 2005–06 and 2015–2016 which is accessible on the Demographic and Health Survey (DHS) website https://dhsprogram.com/information/dataset/India_Standard-DHS_2015.cfm?flag=1 therefore, doesn’t require any ethical approval for the use of data.The National Family Health Surveys (NFHS) are part of the global Demographic and Health Surveys (DHS), conducted by the IIPS (Mumbai), with support from the Ministry of Health and Family Welfare (MoHFW), Government of India and ICF International Inc. [20]. NFHS is a nationally representative, large scale, repeated cross sectional survey in representative samples of households throughout India. NFHS provides important aspects of maternal, child, adolescent and adult health indicators. Details about the NFHS-3 and NFHS-4 sampling designs, tools, and protocols presented in the national reports of NFHS [20] and all relevant information is available in the public domain on http://rchiips.org/NFHS.shtml.

The NFHS survey collected information from a nationally representative sample of 74,369 in NFHS-3 (2005–06) and 112,122 men aged 15–54 years during the period NFHS-4 (2015–16). For the present study, only men aged 15–24 years have been considered. Therefore, a total of 25,538 men in NFHS-3 and 35,712 men aged 15–24 years in NFHS-4 were considered for the analysis. In this study, men have been divided into two age groups as 15–19 years (adolescent boys) and 20–24 years (young men) for comparison purpose.

### Outcome variables

The study used ‘ever had sexual intercourse’, ‘age at first sex’, ‘condom use at first sex’, ‘multiple sexual partners in the past 12 months (having more than one partners)’, ‘relationship with most recent sexual partners’, ‘condom used with most recent sexual partners’ and ‘high-risk sexual behaviour in the past 12months’ as dependent variables. National Family Health Surveys has defined high-risk sexual behaviour as sexual intercourse, in the last 12 months, with someone who is neither a spouse nor a cohabiting partner [20].

### Independent variables

A set of independent variables such as socio-economic characteristics, demographic characteristics and geographical regions of the respondents were included in the analysis. The explanatory variables included in this study are years of schooling, place of residence (rural, urban), regular exposure of media (no, yes), household wealth index (poorest, poorer, middle, richer, richest), religion (Hindu, Muslims and others), membership to social group (Scheduled Castes – SC, Scheduled Tribes – ST, Others), region of residence (Southern, Northern, North-eastern, Central, Eastern and Western), comprehensive knowledge about HIV/AIDS (no, yes), and alcohol use (no, yes).

### Statistical analysis

Bivariate and multivariate analysis was applied to understand the changes in adolescent boys and young men’s sexual behaviour during the last decade according to their socio-economic, demographic characteristics and region of residence. A Binary logistic regression was performed to identify the determinants of risky sexual behaviour among adolescent boys and young men.

Binary logistic regression analysis is useful when the outcome variable has only two categories (0 and 1). The basic form of logistic regression model, which yields the probability of occurring of an event, depicted as:

1 $$ p=\frac{1}{1+e{-}^z}=\frac{e^z}{1+{e}^z} $$

While analysing the association between multi-partner sexual behaviour and selected background characteristics, it was observed that the multi-partner sexual behaviour variable was excess with zero outcome. To overcome this problem we have used a zero inflated Poisson regression model to determine the incidence rate ratio (IRR) of having multiple sexual partners. The zero-inflated Poisson (ZIP) regression model is a modification of the familiar Poisson regression model that allows for an over-abundance of zero counts in the data. The distribution of multi-partner sexual behaviour variable combines the Poisson distribution and the logit distribution. For each observation in the multi-partner sexual behaviour, there are two possible regimes. In one regime the outcome is always a zero count, while in other regime the counts (including zeros) follow a standard Poisson process. Suppose that outcome one occurs with probability π and outcome two occurs with probability 1 - π. Therefore, the probability distribution of the ZIP random variable *y*_*i*_ (multi-partner sexual behaviour) can be written as

$$ {\displaystyle \begin{array}{c}\Pr\ \left({y}_i=j\right)={\pi}_i+\left(1-{\pi}_{\mathrm{i}}\right)\ \exp\ \left(-{\mu}_i\right)\kern1.5em \mathrm{if}\ \mathrm{j}=0\\ {}\Pr\ \left({y}_i=j\right)=\left(1-{\pi}_i\right){\mu}^{yi}\exp\ \left(-{\mu}_i\right)/{\mathrm{y}}_i!\kern1.25em \mathrm{if}\ \mathrm{j}>0\end{array}} $$

Where the outcome variable *y*_*i*_ has any non-negative integer value, μ is the expected Poisson count for the i^th^ individual, and π_i_ is the logistic link function.

In the ZIP regression model, the predictors affecting *π*_*i*_ and μ_i_ may or may not be the same. If the same covariates affect *π*_*i*_ and *μ*_*i*,_ we can write *π*_*i*_ as a function of *μ*_*i*_ to obtain.

$$ \log \kern0.5em \left(\mu i\right)\kern0.5em =\kern0.5em {\sum}_{j=1}^k\kern0.5em \left(\mathrm{x} ij\beta j\right)\kern0.5em \mathrm{and}\kern0.5em \log \mathrm{it}\kern0.5em \left({\pi}_i\right)\kern0.5em \log \kern0.5em \Big(\left(\frac{\pi i}{1-\pi i}\right)\kern0.5em =\kern0.5em -\tau {\sum}_{j=\kern0.5em 1}^k\kern0.5em \left(\mathrm{x}\mathrm{ij}\kern0.5em \beta j\right) $$

Where β*j* (*j* = 1 2 3 …k) is the regression coefficients unknown parameters that are estimated from a set of data.

Statistical analysis was carried out using STATA 13.0 version software. All the analyses were weighted using NFFHS-4 provided sampling weights to account for survey design.

## Results

**Fig. 1:**
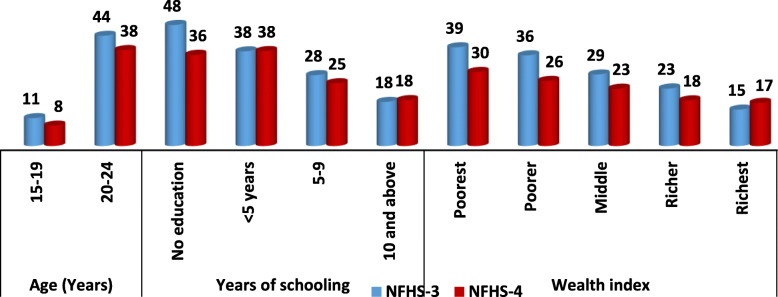
Prevalence of ever had sex among youth (15–24) according to age, education and wealth quintile

**Fig. 2:**
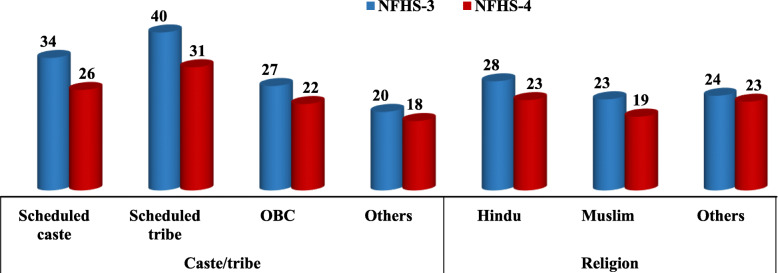
Prevalence of ever had sex among youth (15–24) according to social caste group and religion

**Fig. 3:**
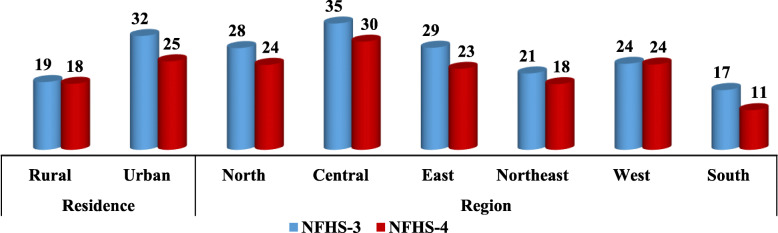
Prevalence of ever had sex among youth (15–24) according to Residence and geographic region

Table 1 shows the prevalence of ever had sexual intercourse among young men (15–24 years) during 2005–06 and 2015–16. Total 6881 in 2005–06 and 7851 adolescent boys and young men in 2015–16 ever had sexual intercourse. Figure 1 shows that ever had sexual intercourse has substantially decreased among adolescent boys (11 to 8%) and young men (44 to 38%) during the last decade. Ever had sexual intercourse has decreased among uneducated young men from 48% to 36% during NFHS-3 to NFHS-4; however, it has slightly increased among higher educated men during the last decade. Although, ever had sexual intercourse is higher among young men residing in urban areas than the rural areas in both the consecutive survey, the prevalence of ever had sexual intercourse decreased from 32% in 2005–06 to 25% in 2015–16 among young men residing in urban areas (Fig. 3). Around three-fifths of young men belonging to SC/ST had ever been sexually active during 2005–06, whereas this proportion decreased in 2015–16 (Fig. 2). In case of socioeconomic status, as wealth index increases ever had sexual activity among adolescent boys and young men decreased in both the consecutive surveys (Fig. 1). A Central region of India shows the highest proportions of adolescent boys and young men involved in sexual activity in both the consecutive surveys than the other geographic regions; however, the percentage has substantially declined in all the geographic areas in India during the last decade (Fig. 3).

**Table 1:** Prevalence of ever had sex among youth (15–24) during NFHS-3 and NFHS-4

	NFHS-3 (2005–06)	NFHS-4 (2015–16)
**No. of Women**	6881	7851
**%**	26.9	22.4

Table 2 shows the transition of mean age at first sex and condom use at first sex among adolescent boys (15–19 years) and young men (20–24 years) in India over the last decade. Results show that the initiation of sexual activity is still early among adolescent boys (16 years) and young men (19 years) over the last decade. Condom use at first sex among adolescent boys (19 to 34%) and young men (14 to 26%) have considerably increased almost double from 2005 to 06 to 2015–16 respectively. Findings evident that condom use at first sex is higher among adolescent boys and young men residing in an urban setting, belonging to the other backward castes (OBC), and who have regular exposure to media than their counterparts in both the survey.

**Table 2:** Mean age at first sex and condom use at first sex among adolescent boys and young men in India

	NFHS-3 (2005–06)				NFHS-4 (2015–16)			
	15–19		20–24		15–19		20–24	
**Background Characteristic**	**Mean age at first sex(years)**	**Condom use at first sex (%)**	**Mean age at first sex (years)**	**Condom use at first sex (%)**	**Mean age at first sex (years)**	**Condom use at first sex (%)**	**Mean age at first sex (years)**	**Condom use at first sex (%)**
**Years of schooling**								
No education	15.7	11.7	18.6	5.4	16.2	30.8	19.1	16.2
< 5	15.9	9.9	19.1	9.5	16.1	34.0	19.1	15.2
5–9	15.7	19.5	19.0	13.8	15.9	30.4	19.3	20.2
10 and above	16.1	24.1	19.4	23.1	16.6	38.1	19.7	34.6
**Place of residence**								
Rural	15.6	13.8	18.8	10.5	16.2	30.5	19.4	21.0
Urban	16.1	34.0	19.4	23.5	16.5	43.6	19.6	37.5
**Religion**								
Hindu	15.7	16.6	19.1	13.4	16.2	31.4	19.4	26.6
Muslim	15.8	25.2	19.2	15.3	16.3	46.9	19.5	21.6
Others	16.3	34.1	18.8	23.2	16.6	45.1	19.5	30.2
**Caste/tribe**								
Scheduled caste	15.6	13.9	19.0	14.6	16.5	36.0	19.5	25.8
Scheduled tribe	16.1	13.7	18.6	7.0	16.2	25.5	19.1	16.0
Other backward class	15.8	14.9	19.0	11.1	16.1	30.8	19.4	25.9
Others	15.9	31.6	19.5	22.3	16.5	45.8	19.8	32.3
**Regular exposure of media**								
No	15.9	12.3	18.7	5.0	16.2	17.9	19.2	14.5
Yes	15.8	19.8	19.1	16.4	16.3	37.8	19.5	28.8
**Wealth index**								
Poorest	15.4	11.7	18.3	5.5	16.0	21.6	19.1	15.6
Poorer	15.6	12.2	18.8	8.8	16.2	29.8	19.3	20.0
Middle	15.8	14.1	19.0	13.8	16.2	30.0	19.5	25.4
Richer	15.9	26.7	19.3	18.7	16.5	44.1	19.7	30.9
Richest	16.3	41.6	19.7	30.7	16.7	56.0	19.9	42.6
**Comprehensive knowledge about HIV/AIDS**								
No	15.7	18.9	19.0	14.1	16.2	30.3	19.5	25.3
Yes	16.0	25.8	19.3	19.9	16.6	40.9	19.6	33.4
**Alcohol Use**								
No	15.7	17.1	19.2	13.0	17.1	31.9	13.0	23.8
Yes	16.0	21.4	18.9	15.9	21.4	39.8	15.9	31.6
**Region**								
North	15.9	29.2	18.9	1.6	16.6	36.0	19.6	28.6
Central	15.4	15.8	18.5	10.7	16.0	30.4	19.2	24.1
East	16.1	10.2	19.2	14.1	16.2	34.0	19.6	21.2
Northeast	16.2	21.0	18.9	11.6	16.4	16.2	19.4	15.2
West	15.7	31.5	19.8	23.6	16.4	38.8	19.4	37.3
South	16.4	18.5	19.7	12.0	16.7	46.5	20.5	19.1
**Total**	15.8	18.5	19.1	14.1	16.3	34.2	19.5	26.1

Adolescent boys and young men belonging to better-off households are more likely to use a condom at first sex in both the survey. Condom used at first sex has increased among adolescent boys (21 to 40%) and young men (16 to 32%), even those who consumed alcohol during the last decade. Condom use at first sex among adolescent boys has rapidly increased during last 10 years in the Eastern and Southern region, although other geographic region also showed the increased prevalence of condom use at first sex from NFHS-3 to NFHS-4.

### Multi-partner sexual behaviour

Table 3 shows the percentage distribution of adolescent boys and young men having multiple sexual partners in the last 12 months and the results of zero-inflated poison regression of having multiple sexual partners in 2005–06 and 2015–16 in India. It is evident that multi-partner sexual behaviour among adolescent boys decreased (15 to 10%) during the last 10 years. The prevalence of multiple sexual partner has declined during the last 10 years among adolescents boys having 10th and above year of schooling (17.2 to 8.6%). In 2015–16, adolescent boys residing in rural setting (7%) are having more multiple sexual partners than the urban setting (11%). An estimated 7 % of adolescent boys belonging to other religion (Christian, Sikhs, Buddhism, Jain, etc.) reported multiple sexual partners in 2015–16 while in 2005–06, this proportion was almost four times higher.

**Table 3:** Percentage distribution of adolescent boys and young men having multiple sexual partners in the last 12 months and results of zero inflated poison regression in India

	NFHS-3 (2005–06)				NFHS-4 (2015–16)			
	15–19		20–24		15–19		20–24	
**Background Characteristics**	**Multiple partners**	**IRR(CI)**	**Multiple partners**	**IRR(CI)**	**Multiple partners**	**IRR(CI)**	**Multiple partners**	**IRR(CI)**
**Years of schooling**								
No education®	8.4		3.7		2.4		6.0	
< 5 years	14.1	1.37(0.90,2.08)	7.7	1.03(0.88,1.20)	15.3	0.74(0.43,1.28)	5.1	1.14(0.95,1.36)
5–9	15.8	1.10(0.78,1.55)	5.3	0.82***(0.72,0.92)	11.8	0.87(0.63,1.20)	5.3	1.04(0.91,1.18)
10 and above	17.2	0.90(0.62,1.31)	5.6	0.58***(0.50,0.66)	8.6	0.97(0.70,1.35)	6.4	0.72***(0.63,0.82)
**Residence**								
Rural®	13.0		5.2		11.1		5.4	
Urban	21.3	0.71***(0.58,0.87)	5.5	0.74***(0.68,0.81)	6.5	0.82**(0.69,0.97)	6.9	0.86***(0.79,0.92)
**Religion**								
Hindu®	15.0		5.0		10.3		5.7	
Muslim	10.7	1.35**(1.04,1.75)	6.4	1.10*(0.98,1.24)	7.7	1.13 (0.89,1.42)	5.0	0.95(0.86,1.05)
Others	22.0	0.83(0.61,1.12)	8.4	1.03(0.90,1.16)	6.9	1.66***(1.31,2.11)	10.5	1.04(0.93,1.16)
**Caste/tribe**								
Scheduled Caste®	13.4		5.8		10.9		6.1	
Scheduled Tribe	9.1	0.92(0.68,1.24)	3.3	1.03(0.90,1.18)	6.5	0.94(0.76,1.16)	5.0	1.002(0.91,1.10)
Other Backward Class	15.9	0.88(0.71,1.08)	5.2	0.94(0.85,1.03)	10.6	0.99(0.83,1.18)	6.7	0.96(0.89,1.04)
Others	19.2	0.70***(0.55,0.89)	6.5	0.97(0.87,1.07)	10.4	0.72***(0.58,0.90)	4.6	0.77***(0.69,0.85)
**Regular exposure of media**								
No®	14.1		3.6		7.0		5.2	
Yes	14.9	0.90(0.69,1.17)	5.8	0.92(0.82,1.03)	10.4	1.19*(0.97,1.46)	6.0	0.90**(0.83,0.99)
**Wealth index**								
Poorest®	12.1		5.4		9.4		3.9	
Poorer	13.0	0.99(0.75,1.31)	5.6	1.09(0.94,1.25)	10.0	0.81**(0.66,0.99)	7.1	1.03(0.93,1.13)
Middle	10.1	0.69**(0.51,0.93)	6.0	0.98(0.85,1.14)	9.5	0.77**(0.62,0.97)	6.4	1.04(0.93,1.15)
Richer	17.3	0.90(0.66,1.22)	4.3	0.87*(0.75,1.02)	12.9	0.80*(0.63,1.02)	5.4	0.98(0.87,1.10)
Richest	29.1	0.80(0.57,1.12)	5.0	0.74***(0.63,0.88)	7.0	0.75**(0.57,0.99)	6.3	1.003(0.88,1.14)
**Comprehensive knowledge about HIV/AIDS**								
No®	14.2		5.0		9.8		5.4	
Yes	16.7	1.67***(1.24,2.25)	6.4	0.97(0.90,1.05)	8.2	0.92(0.70,1.20)	8.2	1.06*(0.99,1.12)
**Alcohol Use**								
No®	10.3		2.6		8.4		4.5	
Yes	23.8	3.61***(2.67,4.87)	9.7	2.04***(1.89,2.19)	13.1	2.30***(1.64,3.23)	8.9	1.59***(1.50,1.70)
**Region**								
North®	17.6		4.9		6.9		5.4	
Central	12.5	1.02(0.80,1.30)	7.4	1.07(0.96,1.20)	11.7	1.77***(1.47,2.13)	7.3	1.17***(1.08,1.27)
East	16.2	0.52***(0.37,0.73)	4.9	0.54***(0.45,0.63)	10.5	0.87(0.68,1.10)	2.5	0.79***(0.71,0.88)
Northeast	10.9	0.45***(0.32,0.62)	4.0	0.58***(0.50,0.66)	6.5	0.52***(0.39,0.70)	3.0	0.65***(0.57,0.74)
West	24.5	0.56***(0.41,0.76)	3.9	0.63***(0.55,0.72)	10.0	1.56***(1.22,1.99)	6.9	1.04(0.93,1.16)
South	2.7	0.15***(0.11,0.22)	3.4	0.43***(0.38,0.48)	1.7	0.17***(0.11,0.27)	7.0	0.42***(0.36,0.48)
**Total**	14.8		5.3		9.8		5.9	

® Reference, *p < 0.10; **p < 0.05; ***p < 0.01; *IRR* Incidence rate ratio; *CI* Confidence interval

The multi-partner sexual behaviour has also decreased among adolescent boys who have regular exposure of mass media 2005–06 (15%) to 2015–16 (10%). A rapid declining pattern was observed in the multiple sexual partner behaviour among adolescent boys belonging to the affluent class of households from 29% in 2005–06 to 7 % in 2015–16. Adolescent boys who have comprehensive knowledge of HIV/AIDS were more likely (IRR = 1.67; *p* < 0.01) to have more than one partner in 2005–06 whereas in 2015–16, they are less likely to report multiple sexual partners. Alcohol-using adolescent boys and young men are more likely to have more than one partner in both the survey, the rate of decrement is also observed over the last one decade.

Regional differences were also observed among adolescent boys and young men. In 2015–16, Central (12%), Eastern (11%) and Western (10.0%) region of adolescent boys shows the higher multi-partner sexual behaviour, whereas in NFHS-3, Northern (18%), Eastern (16%) and Western (24%) region of adolescent boys shows higher multi-partner behaviour than the other geographic regions of India. Zero-inflated Poisson regression results also revealed that adolescent boys belonging to Central (IRR = 1.77; *p* < 0.01), and Western region (IRR = 1.56; p < 0.01) were more likely to have more than one partner in 2015–16 than the other counterparts.

### Relationship status with the most recent sexual partners

Table 4 portrays the status of relationship with the most recent partner among adolescent boys and young men according to their place of residence in 2005–06 and 2015–16 in India. Results show that relationship status with girlfriends/fiancé (57%), commercial sex workers (8.4%), and live-in partner (11%) is higher among adolescent boys residing in urban areas than the young men living in urban areas during 2015–16. It was observed that the percentage has increased in rural areas among adolescent boys having girlfriends/fiancé (29.6 to 48.6%), casual acquaintance (2.3 to 10.6%), commercial sex workers (2.3 to 5.5%) and live-in partner (0.6 to 6%) during the last one decade.

**Table 4:** Relationships Status with the most recent partners among adolescent boys and young men in India

			Spouse	Girlfriend/fiancé	Casual acquaintance	Commercial sex workers	Live-in partner	Others
**NFHS-4 (2015–16)**	**15–19**	Rural	28.7	48.6	10.6	5.5	6.0	0.6
		Urban	16.2	57.2	6.5	8.4	10.9	0.8
		**Total**	25.2	51.0	9.5	6.3	7.4	0.7
	**20–24**	Rural	77.9	15.2	2.4	1.8	2.4	0.3
		Urban	60.2	28.2	4.9	2.2	4.1	0.4
		**Total**	72.5	19.2	3.2	1.9	2.9	0.3
**NFHS-3 (2005–06)**	**15–19**	Rural	44.6	29.6	2.3	2.3	0.6	20.6
		Urban	15.0	54.9	3.1	8.9	3.1	15.1
		**Total**	38.2	35.0	2.5	3.7	1.2	19.4
	**20–24**	Rural	87.3	5.7	0.8	0.9	0.5	4.9
		Urban	75.3	12.7	0.5	3.3	0.9	7.4
		**Total**	84.2	7.5	0.7	1.5	0.6	5.5

Relationships status with most recent sexual partners has also been analysed with the socioeconomic status of the adolescent boys and young men in both the survey (Table 5). Adolescent boys belonging to the poorest quintile (41%) are more likely to have girlfriends/fiancé in 2015–16 than the adolescent boys in 2005–06. However, the culture of girlfriends/fiancé among adolescent boys have considerably increased in across the wealth quintile from 2005 to 06 to 2015–16. During the last 10 years, live-in partner relationships have also increased among adolescent boys, whether they belong to lower socio-economic status of households (0.0 to 6.3%) or higher socioeconomic status (1.4 to 13%) from NFHS-3 to NFHS-4.

**Table 5:** Relationship with most recent partner according to socioeconomic status among adolescent boys (15–19) in India

Socioeconomic Status		Spouse	Girlfriend/fiancé	Casual acquaintance	Commercial sex worker	Live-in partner	Others
**NFHS-4 (2015–16)**	**Poorest**	32.4	41.2	11.1	7.8	6.3	1.2
	**Poorer**	28.8	44.7	12.8	8.0	5.7	0.0
	**Middle**	27.9	54.9	5.1	5.4	6.1	0.7
	**Richer**	18.4	61.3	7.9	3.8	7.1	1.5
	**Richest**	13.2	59.9	8.6	5.3	13.1	0.0
	**Total**	**25.2**	**51.0**	**9.5**	**6.3**	**7.4**	**0.7**
**NFHS-3 (2005–06)**	**Poorest**	45.0	32.2	1.6	1.9	0.0	19.3
	**Poorer**	50.0	24.0	1.7	1.8	1.8	20.8
	**Middle**	41.1	29.5	4.5	2.5	0.6	21.9
	**Richer**	24.4	46.4	1.6	9.4	2.3	15.9
	**Richest**	10.3	60.8	4.0	6.6	1.4	16.9
	**Total**	**38.2**	**35.0**	**2.5**	**3.7**	**1.2**	**19.4**

### Condom use during last sexual intercourse

Overall, condom use during last sexual intercourse among adolescent boys (23 to 69%) and young men (13 to 65%) has rapidly increased over the last one decade (Table 6). It was observed that as the level of education increases, condom use during last sexual intercourse also increased among adolescent boys and young men in both the survey. The prevalence of condom use during last sexual intercourse increased among adolescent boys (37 to 72%) and young men (20 to 67%) from NFHS-3 to NFHS-4 those who have comprehensive knowledge of HIV/AIDS.

**Table 6:** Condom use at last sex with most recent partners among adolescent boys and young men in India

Background Characteristics	NFHS-3 (2005–06)		NFHS-4 (2015–16)	
	15–19	20–24	15–19	20–24
**Years of schooling**				
No education	12.0	5.8	46.8	68.3
< 5 years	8.4	7.7	51.9	49.4
5–9	24.1	11.3	67.7	59.2
10 and above	33.4	23.8	72.0	68.0
**Residence**				
Rural	16.2	9.7	68.3	64.6
Urban	46.3	21.7	69.1	65.7
**Religion**				
Hindu	21.1	12.0	67.1	64.4
Muslim	27.0	12.9	71.9	66.0
Others	38.0	26.2	79.3	72.5
**Caste/tribe**				
Scheduled caste	18.8	12.4	77.1	72.1
Scheduled tribe	17.4	4.6	48.8	67.6
Other backward class	18.9	10.5	68.8	65.3
Others	39.3	21.0	63.7	61.1
**Regular exposure of media**				
No	11.5	5.1	62.5	38.1
Yes	25.2	14.8	69.2	67.7
**Wealth index**				
Poorest	11.5	3.7	62.8	62.5
Poorer	13.9	8.3	69.7	65.8
Middle	18.6	10.7	57.7	60.5
Richer	38.9	17.4	81.6	62.1
Richest	50.7	34.6	68.7	71.3
**Comprehensive knowledge about HIV/AIDS**				
No	21.8	11.5	66.4	64.3
Yes	36.5	19.6	72.2	67.1
**Alcohol Use**				
No	19.7	11.8	68.7	61.0
Yes	28.7	14.3	68.6	72.0
**Region**				
North	28.9	19.8	68.9	65.8
Central	19.9	12.4	66.3	57.6
East	14.2	6.9	66.3	64.7
Northeast	29.3	10.5	48.2	55.1
West	43.0	18.5	68.8	70.7
South	16.2	7.9	90.0	76.4
**Total**	22.7	12.8	68.8	65.1

The results also reported that the prevalence of condom use during last sexual intercourse increased among alcohol-using adolescent boys and young men over the last one decade. Regional differences was also observed in the prevalence of condom use during last sexual intercourse in the last decade, as Southern region of adolescent boys and young men reporting higher prevalence than the other geographic regions of India in 2015–16. Condom use during last sexual intercourse in Central (20% & 12 to 66% & 58%), Eastern (14% & 7 to 66% & 65%) have rapidly increased among adolescent boys and young men from 2005 to 06 to 2015–16 respectively.

### High-risk sexual behaviour in the past 12 months

Table 7 estimated the high-risk sexual behaviour in the past 12 months among adolescent boys and young men in India. Overall, high-risk sexual behaviour has increased among adolescent boys (64 to 70%) and young men (18 to 27%) from 2005 to 06 to 2015–16. The odds of engaging in high-risk sexual behaviour in the past 12 months was higher among adolescent boys (AOR = 1.98; *p* < 0.01), and young men (AOR = 2.74; *p* < 0.01), who are having 10th and above years of schooling than the other counterparts in 2015–16 (Table 8). High-risk sexual behaviour has decreased among adolescent boys residing in urban areas (85 to 74%) during the last decade, while it has increased among young men from 26% in 2005–06 to 38% in 2015–16. The results of the binary logistic regression analysis revealed that adolescent boys and young men residing in the urban setting are significantly two times (*p* < 0.01) and three times (*p* < 0.01), more prone to engage in high-risk sexual behaviour in the past 12 months during 2015–16.

**Table 7:** High-risk sexual behaviour in the past 12 months among adolescent boys and young men in India

Background Characteristics	NFHS-3 (2005–06)		NFHS-4 (2015–16)	
	15–19	20–24	15–19	20–24
**Years of schooling**				
No education	43.3	11.6	59.1	16.1
< 5 years	62.5	13.3	58.4	14.5
5–9	65.3	17.0	67.1	20.7
10 and above	75.4	27.8	74.2	36.8
**Residence**				
Rural	58.0	15.4	67.5	22.0
Urban	85.1	25.8	74.4	38.0
**Religion**				
Hindu	65.8	16.8	69.5	26.8
Muslim	46.6	20.3	68.2	21.0
Others	73.9	35.6	73.4	42.5
**Caste/tribe**				
Scheduled caste	69.6	18.0	76.0	76.0
Scheduled tribe	56.0	14.8	60.7	60.7
Other backward class	59.3	15.3	67.1	67.1
Others	75.1	25.0	79.8	79.8
**Regular exposure of media**				
No	41.4	9.7	49.0	14.1
Yes	69.0	20.3	74.2	30.0
**Wealth index**				
Poorest	57.4	13.6	63.3	15.8
Poorer	52.9	14.0	67.4	22.3
Middle	60.8	17.9	67.7	23.1
Richer	77.3	19.9	77.6	34.6
Richest	89.7	32.1	74.9	43.9
**Comprehensive knowledge about HIV/AIDS**				
No	62.3	18.0	71.8	25.4
Yes	75.3	23.5	71.2	35.1
**Alcohol Use**				
No	60.6	13.3	67.1	23.5
Yes	70.4	25.7	75.0	34.3
**Region**				
North	59.7	19.5	70.6	30.3
Central	67.2	19.6	81.5	32.6
East	46.7	13.2	58.5	12.7
Northeast	74.4	19.4	37.4	14.9
West	80.8	20.3	58.4	33.3
South	76.5	17.8	63.0	19.5
**Total**	63.8	18.1	69.5	26.9

**Table 8:** Factor Associated with high-risk sexual behaviour among adolescent boys and young men in India

Background Characteristics	NFHS-3 (2005–06)		NFHS-4 (2015–16)	
	Adjusted Odds Ratio (CI)		Adjusted Odds Ratio (CI)	
	15–19	20–24	15–19	20–24
**Years of schooling**				
No education®				
< 5 years	1.63(0.64,4.13)	1.08(0.68,1.7)	4.57*(0.81,25.6)	0.95(0.58,1.56)
5–9	1.57(0.75,3.31)	1.38*(0.98,1.96)	1.20(0.55,2.6)	1.24(0.88,1.76)
10 and above	1.81(0.79,4.15)	2.22***(1.52,3.23)	1.98*(0.88,4.46)	2.74***(1.93,3.9)
**Residence**				
Rural®				
Urban	1.79**(1.06,3)	1.30**(1.05,1.61)	2.23***(1.33,3.75)	1.74***(1.47,2.06)
**Religion**				
Hindu®				
Muslim	0.57*(0.3,1.07)	1.53***(1.14,2.05)	1.04(0.54,2.01)	1.12(0.88,1.43)
Others	1.67(0.71,3.92)	1.90***(1.39,2.6)	2.05*(0.96,4.38)	2.10***(1.61,2.74)
**Caste/tribe**				
Scheduled caste®				
Scheduled tribe	0.45**(0.21,0.94)	0.96(0.67,1.36)	0.87(0.48,1.57)	1.16(0.91,1.47)
Other backward class	0.49***(0.29,0.83)	0.76**(0.58,0.98)	0.79(0.48,1.29)	1.03(0.85,1.24)
Others	0.68(0.36,1.31)	0.99(0.76,1.29)	1.68(0.84,3.38)	1.19(0.94,1.51)
**Regular exposure of media**				
No®				
Yes	1.26(0.71,2.23)	1.55**(1.1,2.19)	2.88***(1.78,4.66)	1.62***(1.29,2.04)
**Wealth index**				
Poorest®				
Poorer	1.41(0.78,2.54)	0.73(0.49,1.09)	1.29(0.75,2.23)	1.22(0.96,1.57)
Middle	1.07(0.57,2.01)	0.93(0.63,1.36)	0.82(0.45,1.49)	1.14(0.88,1.47)
Richer	2.29**(1.12,4.7)	0.86(0.58,1.29)	1.18(0.6,2.34)	1.32*(1,1.74)
Richest	2.82**(1.18,6.75)	1.34(0.87,2.06)	1.41**(0.62,3.2)	1.25***(0.93,1.69)
**Comprehensive knowledge about HIV/AIDS**				
No®				
Yes	0.76(0.5,1.16)	0.76(0.5,1.16)	1.02(0.7,1.49)	1.30***(1.13,1.5)
**Alcohol Use**				
No®				
Yes	1.24(0.8,1.93)	2.26***(1.87,2.72)	1.82***(1.18,2.81)	2.38***(2.05, 2.77)
**Region**				
North®				
Central	1.90**(1.05,3.42)	1.14(0.84,1.53)	3.42***(1.99,5.88)	1.61***(1.33,1.94)
East	1.25(0.56,2.82)	0.64*(0.39,1.03)	0.80(0.43,1.47)	0.51***(0.39,0.67)
Northeast	1.39(0.62,3.16)	1.27(0.89,1.83)	0.60(0.26,1.39)	0.86(0.62,1.17)
West	2.91**(1.21,6.98)	1.25(0.9,1.74)	0.62(0.34,1.14)	1.31**(1.02,1.69)
South	3.57**(1.32,9.64)	0.87(0.62,1.23)	2.29(0.46,11.4)	0.60***(0.42,0.85)
**Constant**	0.777	0.088	0.535	0.065

® Reference, *p < 0.10; **p < 0.05; ***p < 0.01; *CI* Confidence intervals

Analysis shows that as wealth index increases, high-risk sexual behaviour increased among adolescent boys and young men in both the surveys, although the magnitude of change has also been reported. The richest quintile of adolescent boys shows the declining trend from 90% in 2005–06 to 75% in 2015–16. The results depicted that adolescent boys and young men having comprehensive knowledge of HIV/AIDS are more involved in high-risk sexual behaviour in both the surveys. Multivariate analysis shows that alcohol-using adolescent boys (AOR = 1.82; *p* < 0.01) and young men (AOR = 2.38; *p* < 0.01) were significantly more likely to engage in high-risk sexual behaviour in 2015–16 than their counterparts.

It was observed that high-risk sexual behaviour among adolescent boys and young men belonging to Northern, Central, and Eastern region has substantially increased from 2005–06 to 2015–16, which was lower in 2005–06 than the other geographical regions of India. The results of the multivariate analysis also revealed that adolescent boys and young men belonging to the Central region are three times (*p* < 0.01) and two times (*p* < 0.01) more likely to engage in high-risk sexual behaviour in the past 12 months in 2015–16 (Table 8).

## Discussion

The present study attempted to assess the changes in sexual behaviour of adolescent boys and young men in India and associated factors using two rounds of NFHS survey. Adolescent sexuality has changed over the last five decades, with adolescents now reaching physical maturity earlier and marrying later. There are several factors which contribute to adolescents’ reproductive health and behaviours. The socio-demographic elements which includes place of residence, family wealth, and own family composition provide the context for adolescent alternatives and decisions; individual characteristics which include academic and modern-day educational and employment repute make a contribution to the human assets that define adolescents’ existent and shape their future [21–24].

The initiation of sexual activity is essential in the transition from adolescence to adulthood [25]. The finding of the study revealed that initiation of sexual activity is still early among adolescent boys (16 years) and young men (19 years) over the last decade in India. This finding indicates that premarital sexual behaviour is common among men and seems to be growing liberalism about sexuality among the youth [6]. This is similar to the findings from others studies conducted in various parts of India [26, 27].

The findings of the study revealed that condom use at sexual debut enhanced the probability of condom use during last sex, and this impact was to a great extent autonomous of the stimulus of stable demographic and individual attributes and proximate attitudinal, social, and relationship factors. This finding is predictable with the possibility that early condom use could help set up an example of condom utilize that conveys forward to resulting sexual activity [28, 29].

Having multiple sexual partners is significantly associated with the risk of sexually transmitted infections (STIs) among adolescents [30]. Findings indicated that adolescent boys were having a higher number of partners than the young men in both the survey rounds, however, multi-partner sexual behaviour among adolescent boys decreased over the last decade. Several studies have also documented that adolescents are more likely to report having multiple sexual partners than adults [30–32]. The major predictor of multiple sexual partners that emerged from the study was consuming alcohol. Alcohol using adolescent boys and young men were significantly more likely to have multiple sexual partners in both the surveys. It may be since adolescent boys and young men get easily influenced by mass media, friends and peer pressure, leading them to experiment in risky lifestyles such as smoking, alcohol intake, drug consumption and sexual activity [13].

In modern India, the live-in relationship is becoming more common among youngsters. With urban India becoming more open-minded and the obvious western influence and students moving out of their homes at an early age, live-in relationships have become even more prevalent [33, 34]. Findings of the study also depicted that the percentage of adolescents having girlfriend, commercial sex workers, and live-in relationship has significantly increased in urban areas from NFHS-3 to NFHS-4. Results revealed that adolescent boys and young men residing in rural areas have also adopted the same culture during the last 10 years. Exposure of mass-media has played a major role in this transformation, giving the youth a level playing field through the Internet as a form of interaction with their metropolitan contemporaries. A noticeable outcome of this is the significant increase in the number of live-in relationships in small cities of India [35].

The risky sexual behaviour places individuals at high risk of sexually transmitted infections (STIs), like HIV/AIDS, and having sex before being mature sufficient to distinguish what makes a healthy relationship. The findings highlighted that adolescent boys who had had at least 10th and above years of education, residing in urban areas, and belonging to the affluent class of households were more likely to engage in high-risk sexual activity than the young men in India. Moreover, the prevalence of high-risk sexual behaviour has significantly increased among both adolescent boys and young men from NFHS-3 (2005–06) to NFHS-4 (2015–16).

Alcohol and sexual activity have a very close and robust relation [36]. Alcohol consumption has regularly been referred as increasing adolescents’ risk of HIV infection [37]. Consistent with the other studies, which are robust in other countries concerning risky sexual behaviour and alcohol consumption [38, 39]. Findings of present study also confirmed a strong and significant relationship that alcohol using adolescent and young men is more likely to engage in high-risk sexual activity in both the consecutive surveys.

These findings revealed that there is an urgent need to concentrate awareness and educational efforts on the early adolescent in India. Early commencement into sexual behaviour among adolescent boys and young men anticipated a more significant level of sexual activity [40]. Additionally, it was also found that early initiation into sex at a younger age was fundamentally connected with having more lifetime sexual partners and the decision of first sexual partner. The existing key interventions to sexual and reproductive health (SRH) of adolescents in India focused on the prevention of disease transmission and handlings high-risk behaviours such as delaying sex debut, reducing the sexual relationship with multiple partners, and condom use [41]. Furthermore, the research on the sexual and reproductive health of adolescent boys has been ignored because these research or programs mainly concentrated on girls. As it is evident, sexual and reproductive health is interdependent among both genders, therefore, to ensure equitable gender roles, attitudes, behaviour, and outcomes we need more research and programs to emphasize young boys and men [2].

According to a 2014 report of Ministry of Health and Family Welfare India, about 21 % of India’s population are adolescents (10–19 years) [42, 43]. Lack of complete and thorough sex-education, inaccessibility and lack of awareness about contraceptives, incorrect sex-education because of early presentation to pornography, etc., ending in unhealthy sexual practices and reproductive ill-health are enormous issues that plague the young. They moreover meddled with an important advancement of a person and community. Keeping in mind the changing pattern of the society in India, the Government of India has drawn out the National Education Policy, 2016, which acknowledges the importance of sex-education in schools for adolescent for wellbeing measures [44]. It is not yet clear if this approach will be actualized this time over. The time has come for the educators to understand that impractical, improper knowledge about sex can be perilous and it is better for the adolescent to be aware and organised.

However, the reality is that sexuality education for adolescents is an exceptionally dubious subjects in India. It is viewed as hostile to Indian qualities, and worries that it may prompt hazardous sexual conduct and wantonness [45]. Consequently, youngsters in India don’t approach thorough sexuality training. Indeed, even among couples, conversations around sex and sexuality seldom occur, as it is taboo [46]. There are no particular instructive educational plans for giving sexuality instruction to class school going youngsters, and it’s excluded inside the advising preparing educational plan. Laws ensuring conceptive rights are not adjusted and there is no particular law on sexual rights in India.

## Conclusions

The study concludes that early sexual debut, lower prevalence of condom use at first sexual experience, and alcohol consumption indicate the dangerous interconnection between such behaviours among adolescent boys over the last decade. Therefore, there is an urgent need to adopt integrated approach of prevention strategies at various levels to generate awareness regarding the potential health hazards of alcohol and premarital sexual relationships that could target multiple forms of risky behaviours of adolescent boys. The concept of live-in relationship has also emerged among rural adolescent boys and young men, and not only among urban youth during the last 10 years. The government of India should fix a legal age to be in a live-in relationship among young population. Emerging evidence shows that Indian demographic health survey should also conduct a special survey on adolescent sexual and reproductive health, which will help in a better understanding of the nature of problems among adolescents in India, leading to their causes and solution.

## Data Availability

It is worth mentioning that the dataset is available in the public domain subject to a prescribed registration and approval process. Requisite permission in accessing and usage of dataset was obtained from the MEASURE-DHS archive.

## Abbreviations

NFHS: National family health Survey
DHS: Demographic Health Survey
AOR: Adjusted Odds Ratio
WHO: World Health Organization
JJ Act: Juvenile Justice Act
POCSO Act: Protection of Children from Sexual Offences Act
GOI: Government of India
HIV: Human Immunodeficiency Virus
AIDS: Acquired Immunodeficiency Syndrome
STDs: Sexually Transmitted Diseases
IIPS: International Institute for Population Sciences
IRR: Incidence rate Ratio
